# Involvement of the Hippo pathway in regeneration and fibrogenesis after ischaemic acute kidney injury: YAP is the key effector

**DOI:** 10.1042/CS20150385

**Published:** 2016-01-26

**Authors:** Jing Xu, Pei-Xue Li, Jun Wu, Yi-Jun Gao, Meng-Xin Yin, Ye Lin, Ming Yang, Dong-Ping Chen, Hai-Peng Sun, Zeng-Bo Liu, Xiang-Chen Gu, Hong-Ling Huang, Li-Li Fu, Hui-Min Hu, Liang-Liang He, Wen-Qing Wu, Zhao-Liang Fei, Hong-Bin Ji, Lei Zhang, Chang-Lin Mei

**Affiliations:** * Division of Nephrology, Kidney Institute of CPLA, Changzheng Hospital, Second Military Medical University, 415 Feng Yang Road, Shanghai 200003, P.R. China; † State Key Laboratory of Cell Biology, Innovation Center for Cell Signaling Network, Institute of Biochemistry and Cell Biology, Shanghai Institutes for Biological Sciences, Chinese Academy of Sciences, 320 Yue-Yang Road, Shanghai 200031, P.R. China; ‡ Memorial Sloan Kettering Cancer Center, New York, NY 10065, U.S.A.; § Division of Nephrology, Central Hospital of TaiAn, TaiAn, Shandong 271000, P.R. China; ║ Division of Nephrology, No. 456 Hospital of PLA, Jinan, Shandong 250031, P.R. China; ¶ Division of Nephrology, Yueyang Hospital, Shanghai 200437, P.R. China; ** Laboratory of Angiogenesis and Neurovascular link, Vesalius Research Center, VIB, Leuven, B-3000, Belgium

**Keywords:** acute kidney injury, chronic kidney disease, fibrogenesis, Hippo pathway, repair, Yes-associated protein (YAP)

## Abstract

Renal tubule cells can recover after they undergo AKI (acute kidney injury). An incomplete repair of renal tubules can result in progressive fibrotic CKD (chronic kidney disease). Studies have revealed the relationship between tubular epithelial cells and kidney fibrogenesis. However, the underlying mechanism remains unclear. Hippo pathway components were evaluated in complete/incomplete repair of I/R (ischaemia/reperfusion) AKI rat models, HK-2 cells and AKI human renal biopsy samples. We found that the expression levels of the Hippo pathway components changed dynamically during kidney regeneration and fibrogenesis in rat models of I/R-induced AKI and human renal biopsy samples. The transcription cofactor YAP (Yes-associated protein) might be a key effector of renal regeneration and fibrogenesis. Our results showed further that YAP might elicit both beneficial and detrimental effects on I/R AKI. After I/R injury occurred, YAP could promote the repair of the injured epithelia. The constant YAP increase and activation might be related to interstitial fibrosis and abnormal renal tubule differentiation. These results indicate that the proper modulation of the Hippo pathway, specifically the transcription cofactor YAP, during repair might be a potent therapeutic target in AKI–CKD transition after I/R injury.

## Abstract (precis)

The Hippo pathway plays a stage-specific role in regeneration and fibrogenesis after ischaemia/reperfusion-induced acute kidney injury. The proper modulation of this pathway might be the key point of transition from acute kidney injury to chronic kidney disease.

## CLINICAL PERSPECTIVES

• The incomplete repair after AKI (acute kidney injury) can cause progressive organ dysfunction, but the underlying mechanism remains unknown. In the present study, we found that the Hippo pathway was closely involved in regeneration and fibrogenesis after ischaemic AKI, and we also identified YAP as a key effector.
• YAP might elicit both beneficial and detrimental effects on I/R AKI. After I/R injury occurred, YAP could promote the repair of the injured epithelia. The constant YAP increase and activation might be related to interstitial fibrosis and abnormal renal tubule differentiation.
• The results provide new insight into the mechanism of AKI–CKD (chronic kidney disease) transition. A proper modulation of the Hippo pathway and specifically the transcription co-factor YAP during repair might be a potent therapeutic target.

## INTRODUCTION

AKI (acute kidney injury) is a set of clinical syndromes characterized by the rapid deterioration of the glomerular filtration rate [1]. After AKI occurs, the kidney possesses profound regenerative potential. It can also recover either completely or incompletely after undergoing regeneration and repair [2,3]. Complete repair leads to the full recovery of the kidney and leaves no evidence of injury. Incomplete repair can cause progressive organ dysfunction and increase the risk of CKD (chronic kidney disease) [4].

Studies have substantiated a direct role for the tubule epithelium in the pathogenesis of the AKI–CKD transition [5,6]. Despite these previous findings, regulatory signalling during the transition should be clarified further. Kidney repair and regeneration after AKI occurs through the phenotypic switch of surviving epithelial cells from a mature quiescent to a proliferative state [5,6]. Repair and regeneration are similar to nephrogenic mesenchyme differentiation during embryonic development [1,10]. This similarity indicates that pathways related to embryonic development may play regulatory roles in regeneration, and malfunction may elicit causal effects on the AKI–CKD transition.

With advances in developmental biology, the signalling pathways essential for organism development have been identified [7]. One important pathway is the Hippo pathway [8]. Originally discovered in *Drosophila*, the Hippo pathway is named after *Drosophila* Hippo kinase [9]. The core component of the mammalian Hippo pathway is a three-step kinase cascade composed of Mst1/2 (mammalian sterile 20-like kinase 1/2), Lats1/2 (large tumour suppressor 1/2) and YAP (Yes-associated protein) [11]. The Hippo signalling pathway is also necessary to co-ordinate cell proliferation, death and differentiation [10,11]. Mutations and the down-regulation of Hippo pathway components, such as Mst1/2 and Lats1/2, have been observed in multiple tumours. The Hippo pathway major downstream effector YAP functions as an oncogene in many cancers [13]. Studies have also revealed the roles of this pathway in heart, liver and intestinal injuries and regeneration [14–20]. Nevertheless, the mechanism by which YAP affects renal regeneration after AKI occurs, specifically the effect on the AKI–CKD transition, remains unknown.

In the present study, we evaluated the expression of core Hippo pathway components and the expression of differentiation and proliferation markers over time in complete/incomplete repair of I/R (ischaemia/reperfusion) AKI rat models. The results indicated that YAP may be a key effector of the Hippo pathway in AKI regulation. *In vitro* overexpression and RNAi studies revealed proliferative and pro-fibrotic dual-functional effects of YAP on HK-2 cells. Furthermore, we used digitoxin, a YAP WW domain modulator identified through *in silico* analysis by Sudol et al. [21], to increase YAP activity *in vitro* and *in vivo*. We found that digitoxin elicited pro-fibrotic and proliferative effects in the I/R AKI rat model. We measured the expression of YAP in AKI human renal biopsy samples. YAP protein levels in the cytoplasm and the nucleus increased in regenerative and poorly differentiated renal tubules compared with the control group. The results indicate that the Hippo pathway is involved in the regeneration and fibrogenesis stages after acute I/R injury occurs. In these stages, YAP may be the main effector, providing a potential therapeutic target of the AKI–CKD transition.

## MATERIALS AND METHODS

### Induction of acute kidney injury in rats

Male Sprague–Dawley rats weighing 200–250 g were purchased from the Shanghai SLAC Laboratory Animal Co., Shanghai Laboratory Animal Center. The rats were housed in a specific pathogen-free environment at the Animal Center of the Second Military Medical University at optimal temperature with a 12 h light/12 h dark cycle. The rats were also provided free access to water and standard rat chow. Animal experiments were performed in strict accordance with the animal use protocol approved by the Institutional Animal Care and Use Committee of the Second Military Medical University.

The rats were randomly assigned and anaesthetized intraperitoneally with a ketamine (50 mg/kg) and xylazine (5 mg/kg) mixture. The rats were then placed on a heating table (37°C) to maintain constant body temperature during surgery. Through a midline incision, mild or severe ischaemia was induced by clamping the dissected bilateral renal arteries with non-traumatic microvascular clips (Kang Wei Medical Instrument Co.) for 30 or 45 min as reported previously [22–24]. The clamps were released, and reperfusion was confirmed visually. Sham operations were performed by exposing bilateral renal arteries without inducing ischaemia. During ischaemic interval and anaesthesia, the rats were maintained on warm heating pads (37°C) to maintain body temperature. Pre-warmed saline (3 ml; 37°C) was instilled intraperitoneally as volume supplement before the abdomen was closed in two layers.

The rats were anaesthetized at the indicated times after ischaemia occurred and were sacrificed at 0 h, 12 h, 24 h, 48 h, 72 h, 5 days, 7 days, 14 days and 4 weeks (*n=* 5). The left kidneys were immediately perfused with PBS from the left ventricle, quickly removed and processed for histological evaluation, protein extraction or RNA extraction. Sham operation groups were set at 24 h, 48 h, 5 days, 14 days and 4 weeks (*n=* 3).

### Renal function

A blood sample from each animal was extracted from the vena cava after the rats were killed. Serum blood urea nitrogen and creatinine levels were determined (at the Di-An Medical Laboratory Center, Shanghai).

### Renal histology and immunohistochemistry

The kidneys were removed and fixed in 4% (w/v) paraformaldehyde, embedded in paraffin and cut into 2 μm sections. Kidney sections were stained with H&E (haematoxylin and eosin) and PAS (periodic acid–Schiff) for histopathological examination. Sirius Red, Masson's trichrome and monoclonal anti-mouse αSMA (α-smooth muscle actin) (Sigma, 1:5000 dilution) stains were used to assess collagen. IHC (immunohistochemistry) was performed as described previously [22,23,25]. In brief, the sections were deparaffinized and rehydrated. Endogenous peroxidase was inactivated by incubating in 3% H_2_O_2_ for 15 min. The sections were incubated in a blocking solution at 37°C for 15 min and in primary antibody overnight at 4°C. The following antibodies were used: monoclonal rabbit anti-YAP (Cell Signaling Techno-logy, 1:100 dilution) and anti-vimentin (Cell Signaling Technology, 1:100 dilution), rabbit anti-AQP1 (aquaporin 1) (Millipore, 1:200 dilution), rabbit anti-megalin (Abcam, 1:200 dilution), rabbit anti-pSmad2/3 (Santa Cruz Biotechnology, 1:5000 dilution), rabbit anti-E-cadherin (epithelial cadherin) (Santa Cruz Biotechnology, 1:100 dilution), and mouse anti-PCNA (proliferating-cell nuclear antigen) (Cell Signaling Technology, 1:4000 dilution). On the following day, the sections were washed three times with TBST (0.1%) and incubated with a secondary antibody at 37°C for 15 min. Positive staining was consecutively revealed by horseradish peroxidase-labelled streptavidin and diaminobenzidine substrate. Nuclei were counterstained with haematoxylin. In the control group, a section was stained with secondary antibody only or without antibodies.

### Renal semi-quantitative morphometric evaluation

The sections from the corticomedullary area of each kidney were graded in terms of the severity of interstitial fibrosis: 0, no evidence of interstitial fibrosis; 1, <10% involvement; 2, 10% to <25% involvement; 3, 25% to <50% involvement; 4, 50% to <75% involvement; and 5, >75% involvement. The score of each section was recorded as the mean for ten random fields per section at magnification of ×40 [26,27].

The distribution and expression of cytosolic and nuclear YAP in the corticomedullary region were evaluated as the mean for ten random fields per section at magnification of ×40: 1 (+/−), >25% involvement; 2 (+), 25% to <50% involvement; 3 (++), 50% to <75% involvement; and 4 (+++), >75% involvement.

### Cell culture and treatment

The human HK-2 proximal tubule cell line (CRL-1571, A.T.C.C., Manassas, VA, U.S.A.) was cultured in the base medium K-SFM supplemented with EGF (5 ng/ml epidermal growth factor), BPE (50 μg/ml bovine pituitary extract) and 1% (v/v) FBS [22,24]. Cells were seeded at 10^5^ cells/ml before the experiment was conducted and grown until 90% confluent. In other experiments, HK-2 cells were incubated with 0.1, 1 or 10 μM digitoxin for 24 h and then collected using RIPA buffer. DMSO was added as control.

### Overexpression or knockdown of YAP through lentivirus infection

YAP overexpression or YAP knockdown was performed. In this procedure, recombinant lentiviruses were produced by transiently transfecting HEK (human embryonic kidney)-293T cells (2×10^5^/ml) with the indicated plasmids along with the packaging plasmid VSVG (vesicular stomatitis virus glycoprotein) and Δ8.2 in accordance with the calcium phosphate precipitation method [28]. The following plasmids were used: pCDH-puro-CMV-FLAG, pCDH-puro-YAP, pCDH-puro-YAP S127A and pCDH-puro-YAP 5SA (provided by Professor Qun-Ying Lei, Fudan University, Shanghai, China), and pLKO.1-puro-Luc, pLKO.1-puro-shYAP-1 and pLKO.1-puro-shYAP-2 (provided by Professor Hong-Bin Ji, Shanghai Institute of Biochemistry and Cell Biology, Shanghai, China). Plasmid DNA (5 μg) was transfected per 6 cm diameter dish. Supernatants were harvested after 48 h and filtered through a 0.45 μm pore size filter. HK-2 cells (10^5^/ml) were subsequently infected using the same volume of the supernatants supplemented with 1% (v/v) FBS and selected with 2 μg/ml puromycin after 48 h of incubation.

### Cell proliferation assay

The cells were plated in triplicate wells in a 96-well plate at 10^3^ cells/well and cultured under normal conditions to evaluate the effect of YAP interference and overexpression on HK-2 proliferation. The daily number of metabolically active mitochondria and viable cells was determined at *D*_490_ by using Thiazolyl Blue Tetrazolium Bromide (Amresco) at a final concentration of 1 mg/ml.

### Quantification of mRNA through real-time RT (reverse transcription)–PCR

Total RNA was extracted from the cells or tissues with TRIzol reagent (Invitrogen). RNA (1 μg) from each sample was reverse-transcribed into first-strand cDNA by using ReverTra Ace qPCR RT Master Mix with gDNA Remover (Toyobo). cDNAs were then subjected to real-time PCR analysis using a CFX96 Real-Time PCR system (Bio-Rad Laboratories) with a SYBR Green Master PCR mix (Toyobo). The primers used in this procedure are listed in Table 1. All of the samples were amplified in duplicate. Each experiment was repeated independently three times. Relative gene expression was converted using the 2^−ΔΔ^*^C^*^T^ method against the internal control GAPDH (glyceraldehyde-3-phosphate dehydrogenase).

**Table 1:** Primers for real-time PCR

Gene (human)	Primer sequence	Length (bp)
*COL1A1* (collagen type 1α1)	Forward, 5′-AGGGCCAAGACGAAGACATC-3′	227
	Reverse, 5′-GTCGGTGGGTGACTCTGAGC-3′	
*COL3A1* (collagen type 3α1)	Forward, 5′-A AGGGCAGGGAACAACT-3′	143
	Reverse, 5′-GATGAAGCAGAGCGAGAAG-3′	
*COL4A1* (collagen type 4α1)	Forward, 5′-CTGCCTGGAGGAGTTTAGAA-3′	82
	Reverse, 5′-GCTGTAAGCGTTTGCGTAGTA-3′	
*CTGF* (connective tissue growth factor)	Forward, 5′-TGGCTTTAGGAGCAGTGGG-3′	135
	Reverse, 5′-CTACAGGCAGGTCAGTGAGCA-3′	
*VIM* (vimentin)	Forward, 5′-AGGAAATGGCTCGTCAC-3′	114
	Reverse, 5′-AGGTGGCAATCTCAATGTC-3′	
*TGFB1* (transforming growth factor β)	Forward, 5′-ACTACGCCAAGGAGGTCA-3′	83
	Reverse, 5′-AGCAACACGGGTTCAGGTA-3′	
*AQP1* (aquaporin 1)	Forward, 5′-CCATTTAGAGGGTGAAGGA-3′	115
	Reverse, 5′-CTGACAAGAGGGAGTAGAGAA-3′	
*CDH16* (kidney-specific cadherin)	Forward, 5′-AGGGGAGACACAGAAGGGACT-3′	122
	Reverse, 5′-ACCACCACCACCACCTCAT-3′	
*GGT1* (γ-glutamyltransferase 1)	Forward, 5′-GTTTGTGGATGTGACTGAGG-3′	118
	Reverse, 5′-GAACTCGGGCTTGTAGTAGG-3′	
*YAP1* (Yes-associated protein 1)	Forward, 5′-AACCGTTTCCCAGACTACCT-3′	234
	Reverse, 5′-GCTCCTCTCCTTCTATGTTCA-3′	
*GAPDH* (glyceraldehyde-3-phosphate dehydrogenase)	Forward, 5′-GGAAACTGTGGCGTGATG-3′	285
	Reverse, 5′-TGGGTGTCGCTGTTGAAG-3′	

### Western blot analysis

Renal tissue from the corticomedullary area or HK-2 cells was lysed and denatured at 100°C for 5 min in a SDS buffer and separated by 8%, 10% or 12% PAGE gels. The proteins were transferred on to a PVDF membrane, blocked for 1 h with 5% (w/v) dried non-fat skimmed milk powder in TBST (TBS containing 0.1% Tween 20) and probed with the indicated antibodies. The following primary antibodies were used: rabbit antibodies against YAP, pYAP, Mst1, pMst1, vimentin (Cell Signaling Technology, 1:1000 dilution), αSMA (Abcam, 1:1000 dilution), AQP1 (Millipore, 1:1000 dilution) and CTGF (connective tissue growth factor) (Santa Cruz Biotechnology, 1:400 dilution); and mouse antibodies against PCNA (Cell Signaling Technology, 1:2000 dilution) and Na^+^/K^+^-ATPase (DSHB, 1:1000 dilution). Horseradish peroxidase-conjugated secondary antibodies were applied, and ECL (Pierce) was conducted to detect proteins. A GAPDH-specific antibody (Sigma) was used for loading controls on stripped membranes. Quantification of immunoblots compared the relative ratio of the gray value of pYAP, YAP and pYAP/YAP at all time points with the controls. The results were measured using ImageJ software (NIH) and normalized to GAPDH.

### Human kidney tissue study

The use of human tissues was approved by the Institutional Review Board of Shanghai Changzheng Hospital. All materials were retrieved from renal biopsy samples of hospitalized patients in the Department of Nephrology, Changzheng Hospital, from 1 January 2012 to 30 May 2014. All patients provided written informed consent. Patients who suffered from AKI, but without underlying chronic kidney diseases or glomerular nephritis were recruited to examine YAP expression in their biopsy samples. Dedifferentiated status was also confirmed by positive vimentin staining of tubular epithelial cells. The demographic information of all patients was collected. The patients were categorized into the regenerating group (<30 days) and the fibrogenesis group (≥30 days) according to the days of biopsy from the time of onset. The regenerating group was divided further into mild and dialysis subgroups according to whether dialysis was received during treatment. In the same period, ten patients who were diagnosed with minor lesions or mild mesangial proliferation without tubular interstitial injury in renal pathology were randomly chosen as control subjects.

### Statistical analysis

Results are expressed as means±S.E.M. For normally distributed data, differences within groups were evaluated using ANOVA, and differences between groups were evaluated using Tukey's post-test. Student's *t* test was performed between two groups. For data that were not normally distributed, analyses between groups were evaluated by a Kruskal–Wallis *H* test, and differences within groups were evaluated. The Mann–Whitney *U*-test was conducted between two groups. *P*<0.05 was considered significant.

## RESULTS

### Rat models of complete and incomplete repair of I/R-induced AKI

**Figure 1:**
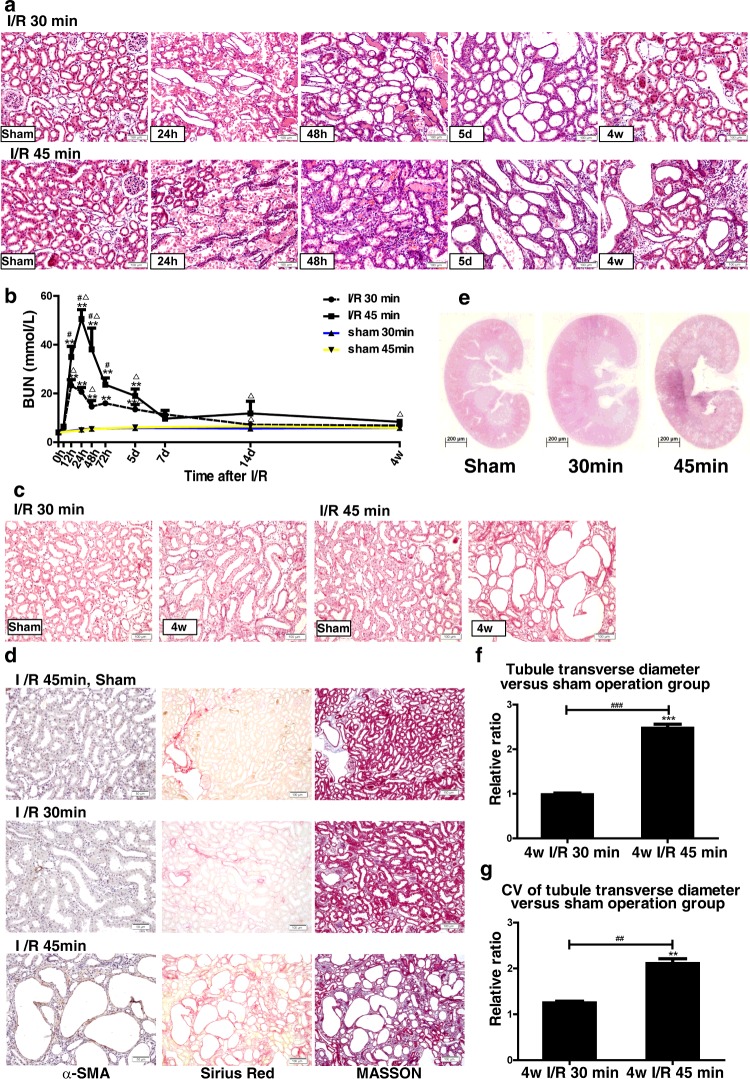
Renal function and pathology of complete and incomplete repair in I/R AKI models (**a**) H&E staining (×200 magnification) of AKI models over time after I/R (*n*=3 rats in each group). Scale bar, 100 μm. (**b**) Changes in serum blood urea nitrogen (BUN) over time in AKI rat models, including mild and severe I/R. **P<0.01 for I/R (*n*=5) compared with control (*n*=3); ^Δ^*P*<0.05 for I/R (*n*=5) compared with sham (*n*=3); ^#^*P*<0.05 for I/R 45 min (*n*=5) compared with 30 min (*n*=5). Results are means±S.E.M. (**c**) PAS staining (×200 magnification) of I/R AKI models after 4 weeks of reperfusion (*n*=3 rats in each group). Scale bar, 100 μm. (**d**) Fibrosis analysis of the I/R AKI models after 4 weeks of reperfusion (*n*=3 rats in each group). Left: immunostaining of αSMA (×200 magnification). Scale bar, 50 μm. Middle: Sirius Red staining showing collagen deposition with red appearance (×100 magnification). Scale bar, 100 μm. Right: Masson's trichrome staining (×100 magnification) showing fibrosis as blue appearance. Scale bar, 100 μm. (**e**) H&E staining of the longitudinal sections from the kidneys subjected to 4 weeks of reperfusion (*n*=3 rats in each group). Scale bar, 200 μm. (**f**) Ratio of the diameters of transverse tubules in the I/R 30 and I/R 45 min groups after 4 weeks of reperfusion compared with sham operation group (*n*=3 rats in each group, 100 tubules in each rat). Results are means±S.E.M. (**g**) Ratio of the coefficient of variation (CV) of the diameters of transverse tubules in I/R 30 and I/R 45 min groups after 4 weeks of reperfusion after I/R compared with sham operation group (*n*=3 rats in each group, 100 tubules in each rat). Results are means±S.E.M.

To investigate the mechanisms of the AKI–CKD transition and the possible function of Hippo signalling in renal regeneration after AKI, we mimicked the complete and incomplete repair of I/R-induced AKI. We constructed two rat models of I/R AKI by changing the time of renal ischaemia from 30 to 45 min to investigate the effects of moderate and severe renal injury. During reperfusion, changes in renal function and morphological characteristics were monitored over time (Figures 1a and 1b). The rats with severe renal injury (I/R 45 min) showed a more significant increase in serum blood urea nitrogen than those with moderate injury (I/R 30 min; Figure 1a). After 4 weeks of reperfusion, kidneys from the I/R 30 min group almost fully recovered from initial injury. However, abnormal differentiation of renal tubules (Figure 1c) and significant tubular interstitial fibrosis (Figure 1d) were observed in rats with severe ischaemia. The longitudinal sections of the 4-week-reperfused kidneys stained with H&E showed numerous dilated tubules in the I/R 45 min group. The dilated tubules were mainly located in the inner cortex and in the outer medulla (Figure 1e), with an increase in the median diameter of the transverse tubule and an increase in the coefficient of variation (Figures 1f and 1g). Multiple poorly differentiated tubules with flattened epithelia, loss of brush border and widened interstitium were observed in I/R 45 min AKI kidneys by PAS staining (Figure 1c). More severe collagen deposition was detected in the I/R 45 min kidneys than in the I/R 30 min group and the sham operation control group, as shown by Sirius Red, Masson's trichrome and αSMA staining (Figure 1d). Thus we constructed two rat models and successfully induced largely reversible mild and moderate AKI and severe AKI with incomplete recovery and persistent renal fibrosis by changing the length of time of renal pedicle clamping.

### Hippo signalling participates in renal regeneration after AKI

**Figure 2:**
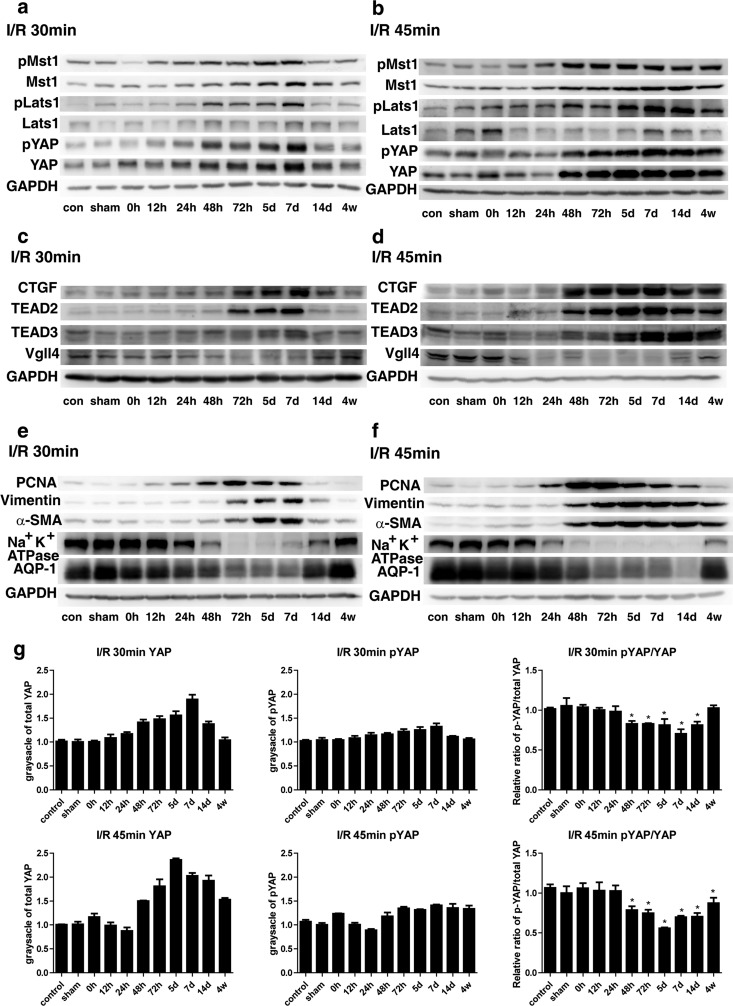
Changes in expression of the key Hippo pathway components and differentiation proteins over time (**a**) Western blot of Mst1/pMst1, Lats1/pLats1 and YAP/pYAP in I/R 30 min rats (*n*=3). (**b**) Western blot of Mst1/pMst1, Lats1/pLats1 and YAP/pYAP in I/R 45 min rats (*n*=3). (**c**) Western blot of CTGF, TEAD2, TEAD3 and Vgll4 in I/R 30 min rats (*n*=3). (**d**) Western blot of CTGF, TEAD2, TEAD3 and Vgll4 in I/R 45 min rats (*n*=3). (**e**) Western blot of differentiation and dedifferentiation markers in I/R 30 min rats (*n*=3). (**f**) Western blot of the differentiation and dedifferentiation markers in I/R 45 min rats (*n*=3). (**g**) Quantification of immunoblots of YAP and pYAP and relative ratio of pYAP to total YAP in 30 and I/R 45 min rats normalized to GAPDH (*n*=3). con, control.

To investigate whether Hippo signalling functions in renal regeneration after AKI, we evaluated the expression of key Hippo pathway components through protein analysis of the inner cortex and the outer medulla. We found that Mst1, Lats1 and YAP expression significantly increased in kidneys of both models after repair (≥48 h; Figure 2a) [1]. The phosphorylation levels of Mst1, Lats1 and YAP (pMst1, pLats1 and pYAP) were also enhanced in both models during recovery (Figure 2b). A constant increase in these proteins and a constant up-regulation of their phosphorylation status were also detected in rats with severe ischaemia during reperfusion. However, this increase returned to levels similar to those in the sham operation and control groups in rats with mild ischaemia. Quantification of immunoblots showed that the increase in YAP after injury was more prominent in the I/R 45 min group than in the I/R 30 min group (Figure 2g). The relative pYAP/total YAP ratio initially decreased at 48 h. This decrease remained significant at 4 weeks in the I/R 45 min group, indicating that YAP activation increased in severe renal injuries after repair and regeneration occurred.

**Figure 3:**
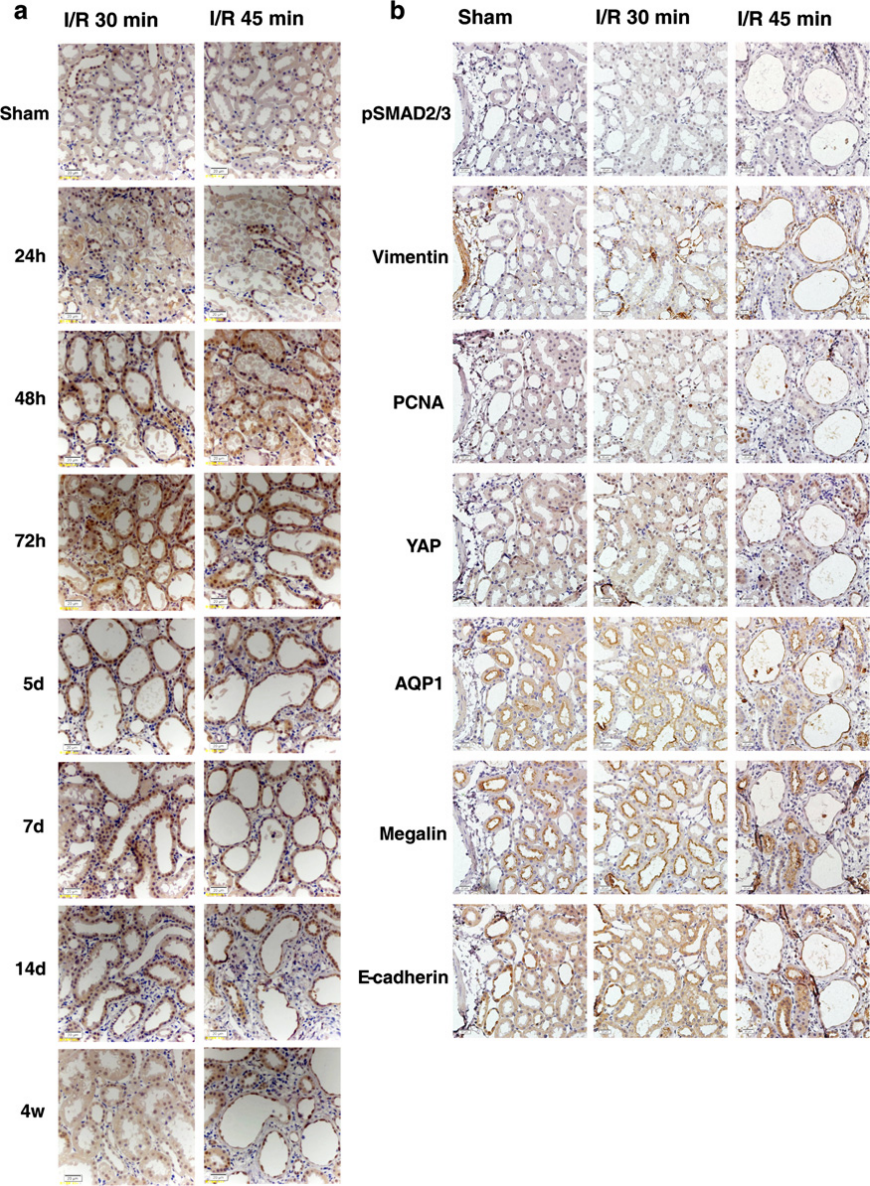
Immunohistochemical staining of YAP and immunostaining of serial sections of YAP co-expressed with differentiation markers (**a**) Immunostaining of YAP (×400 magnification) over time after I/R in 30 and 45 min groups. Scale bar, 20 μm. (**b**) Immunostaining of serial sections of YAP co-expressed with differentiation markers (×400 magnification) over time after I/R in 30 and 45 min groups. Scale bar, 20 μm.

YAP was subjected to IHC staining to detect YAP distribution in kidney tissue sections and to verify the findings shown in Figure 2. YAP was mainly located in tubular epithelial cells and was evenly distributed in the cytoplasm and the nuclei (Figure 3a). In a few cases, YAP was detected in the renal interstitium. At 4 weeks after reperfusion, the YAP expression in the kidneys with mild I/R AKI returned to sham operation levels. However, YAP was highly expressed in the cytoplasm and nuclei of tubular epithelial cells of the poorly differentiated tubules in the kidneys with severe I/R AKI. This result indicated the hyperactivation of YAP protein. YAP is the main effector of the Hippo pathway. We therefore verified whether the expression of YAP activity-related proteins was altered (Figures 2c and 2d). CTGF is one of the commonly reported YAP downstream targets and critical regulatory factors in kidney fibrosis [29]. TEAD2 (TEA domain family member 2) and TEAD3 are TEAD/TEF (transcriptional enhancer factor) transcription factors and well-characterized YAP partners [9,12,13]. Vgll4 (vestigial-like protein 4) is another Hippo pathway member that competes with YAP in terms of binding to TEADs [30,31]. Western blot analyses revealed that CTGF, TEAD2 and TEAD3 expression changed similarly to YAP expression. However, Vgll4 expression changed in a different pattern. The Vgll4 level remained significantly decreased at 4 weeks compared with the sham operation and blank control groups. These results indicate the role of the Hippo pathway in the pathophysiological process of AKI, particularly during the repair stage. Therefore Hippo signalling may regulate renal regeneration after AKI occurs, and the changes in the irreversible protein levels of the Hippo pathway components may induce persistent renal fibrosis.

### Change in differentiation markers correlates with YAP protein levels

To determine the underlying mechanism of renal repair after I/R-induced AKI, we evaluated further the expression of several differentiation markers (Figures 2e and 2f). The dedifferentiation markers vimentin, αSMA and PCNA showed changes in protein levels similar to YAP during recovery in both rat models (compare Figures 2e and 2f with Figures 2a and 2b). The protein levels of markers such as Na^+^/K^+^-ATPase and AQP1 indicated that quiescent cells decreased during repair (Figures 2e and 2f). In the I/R 30 min group, the expression of Na^+^/K^+^-ATPase and AQP1 returned to sham operation and blank control levels after 4 weeks of reperfusion (Figure 2e), whereas in the I/R 45 min group, the expression of these two differentiation markers of mature cells was lower than that of the control groups (Figure 2f). Therefore Hippo signalling may regulate renal regeneration and cell redifferentiation after AKI.

We also evaluated the distributions of dedifferentiation markers and quiescent tubular markers with YAP co-expression in poorly differentiated tubules (Figure 3b). YAP showed a similar expression pattern to vimentin, pSmad2/3 and PCNA in the poorly redifferentiated tubules in the corticomedullary areas of the I/R 45 min models. By contrast, the expression of the quiescent tubular cell markers including megalin, AQP1 and E-cadherin in the YAP-positive tubules was decreased or absent (Figure 3b).

### YAP is essential for cell proliferation and pro-fibrotic functions in HK-2 cells

**Figure 4:**
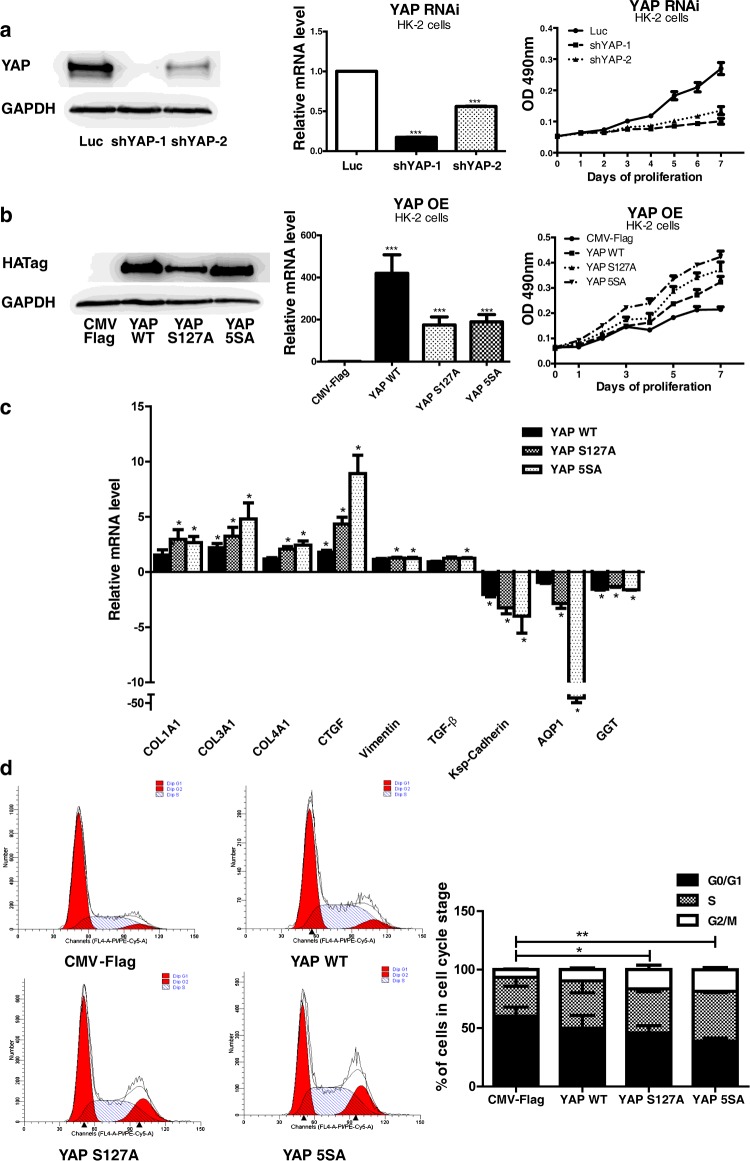
Analysis of YAP overexpression and RNAi in HK-2 cells (**a**) YAP RNAi slowed the proliferation of HK-2 cells. Results are means±S.E.M. (**b**) YAP overexpression (OE) promoted the proliferation of HK-2 cells. Results are means±S.E.M. (**c**) Quantification of mRNA levels of pro-fibrogenic and differentiation genes in YAP-overexpressing HK-2 cells. The mRNA levels are presented as mean±S.E.M. fold induction over the empty vector-transfected control. *P<0.05 compared with control. (**d**) Cell cycle analysis through propidium iodide staining of YAP-overexpressing HK-2 cells. Results in the histogram are means±S.E.M. For comparison of G_2_/M phases, *P<0.05 compared with control; **P<0.01 compared with control. CMV, cytomegalovirus; WT, wild-type.

To evaluate the *in vitro* function of YAP in kidney tubule epithelial cells, we overexpressed or knocked down YAP in HK-2 cells. We found that YAP overexpression significantly promoted, whereas knockdown inhibited, HK-2 cell proliferation (Figures 4a and 4b). As YAP increased, the mRNA level of fibrotic proteins including type I, III and IV collagen increased (Figure 4c). CTGF also increased, indicating that kidney fibrosis was promoted. Conversely, the mRNA levels of mature tubule cell markers including kidney-specific cadherin, AQP1 and γ-glutamyltransferase were reduced (Figure 4c). The up-regulation of YAP induced an increase in G_2_/M- and S-phase and a decrease in G_0_/G_1_-phase HK-2 cells (Figure 4d). An increase in G_2_/M-phase proximal renal tubular cells mediates kidney fibrosis after acute injury [22]. The YAP mutants S127A and 5SA, which escape cytoplasm retention and exhibit hyperactivity, elicited more prominent effects than wild-type YAP (Figures 4c and 4d). These results indicate that an increase in YAP activity not only promotes cell proliferation but also enhances pro-fibrotic effects.

### YAP up-regulation by digitoxin treatment promotes proliferation and fibrogenesis during repair and regeneration of AKI

**Figure 5:**
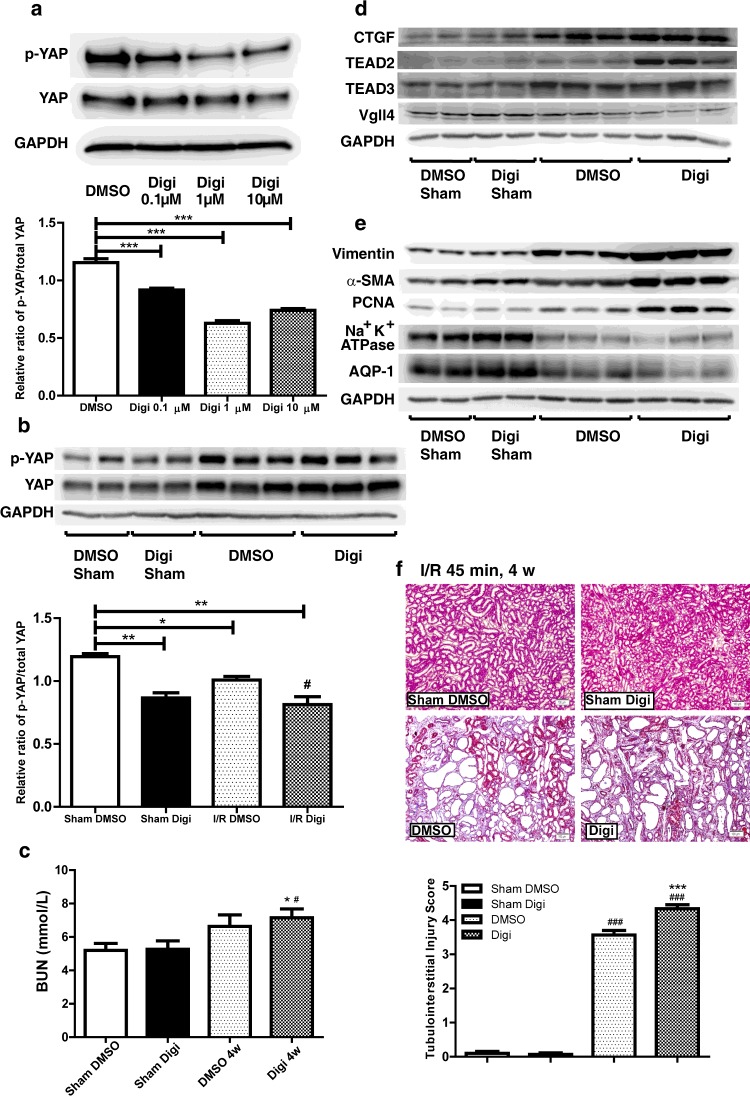
YAP agonist treatment *in vitro* and *in vivo* in the I/R 45 min AKI models (**a**) Western blot of the pYAP/YAP ratio in HK-2 cells with an increased gradient of digitoxin concentration after 24 h of incubation. DMSO was added as control. Quantification of immunoblots of the pYAP/YAP ratio. Results are means±S.E.M. ***P<0.001 compared with control. (**b**) Western blot of pYAP and YAP in DMSO- and digitoxin-treated I/R 45 min rat models 4 weeks after reperfusion. Quantification of immunoblots comparing pYAP/YAP ratio between groups. Results are means±S.E.M. *P<0.05 compared with DMSO-treated sham operation group (*n*=3); **P<0.01 compared with DMSO-treated sham operation group (*n*=3). (**c**) Serum blood urea nitrogen (BUN) after I/R injury followed by DMSO or digitoxin treatment for 4 weeks. Results are means±S.E.M. **P<0.01 for digitoxin-treated I/R (*n*=4) and sham operation (*n*=3) compared with DMSO-treated sham operation (*n*=3); *P<0.05 for DMSO-treated I/R (*n*=3) compared with sham operation (*n*=3); ^#^*P*<0.05 for digitoxin-treated I/R (*n*=4) compared with DMSO-treated I/R (*n*=3). (**d**) Western blot of CTGF, TEAD2, TEAD3 and Vgll4 (*n*=3). (**e**) Western blot of the differentiation and dedifferentiation markers (*n*=3). (**f**) Masson's trichrome staining (×100 magnification) showing fibrosis as blue appearance (*n*=3). Scale bar, 100 μm. Quantification results are means±S.E.M. Digi, digitoxin.

We regulated the YAP expression levels and found that YAP promoted cell proliferation and the expression of pro-fibrotic-related genes, as well as the percentage of HK-2 cells in G_2_/M- and S-phase. We confirmed these findings in I/R-induced AKI rat models to determine the underlying mechanism. On the basis of the results of the *in silico* analysis reported by Sudol et al. [21], the cardiac glycoside digitoxin binds to several residues lining the hydrophobic groove within the WW domain of YAP, including Tyr^188^, Leu^190^, Thr^197^ and Trp^199^, which are critical to the binding of canonical PPXY ligands. Therefore digitoxin can be used to promote the nuclear translocation of YAP to increase YAP activity. As the digitoxin concentration increased, the relative ratio of pYAP to total YAP in HK-2 cells decreased in a partially dose–response manner after 24 h of incubation (Figure 5a). Compared with the DMSO-treated group, the relative pYAP/total YAP ratio in rat kidneys also decreased after 4 weeks of reperfusion when digitoxin was injected intraperitoneally after AKI was induced (Figure 5b). We also evaluated the expression of YAP-related proteins (Figure 5c). The CTGF, TEAD2 and TEAD3 protein levels increased further in the digitoxin-treated group. In contrast, the Vgll4 protein levels decreased further after treatment was administered. The dedifferentiation markers vimentin, PCNA and αSMA changed in a similar trend to YAP (Figure 5d). The expression of tubular epithelial markers including Na^+^/K^+^-ATPase and AQP1 also decreased. Masson's trichrome analysis showed more severe renal interstitial fibrosis in the digitoxin-treated group after I/R AKI than in the DMSO-treated group and the sham operation control group (Figure 5e). This result indicates that a decrease in the relative pYAP/total YAP ratio and an increase in YAP activation could prevent the redifferentiation of dedifferentiated renal tubular cells and promote proliferation and fibrogenesis during repair and regeneration of AKI, which might be a key contributor to the AKI–CKD transition.

**Table 2:** Demographic features of all AKI patients and control subjects Data are expressed as number (%), mean (with S.D.) or median (with range) as appropriate. eGFR, estimated glomerular filtration rate.

			AKI		
			Regenerating		
Characteristic	Total	Control	Mild	Dialysis	Fibrogenesis
Number	43	10	15	8	10
Gender					
Male	24	6	12	5	1
Female	19	4	3	3	9
Age	48 (15–73)	45 (23–60)	43 (15–66)	42.5 (29–73)	57 (38–69)
Aetiology					
Renal ischaemia	11	0	3	5	3
Nephrotoxic drug	3	0	1	0	2
Ischaemia or drug	14	0	7	2	5
Rhabdomyolysis	5	0	4	1	0
Minimal change	7	7	0	0	0
Mild mesangial proliferation	3	3	0	0	0
Time of biopsy after onset	–	–	8.5 (2.7)	11.1 (5.6)	42.3 (14.1)
eGFR (Cockcroft–Gault)	25.2 (3.71–106.6)	88.45 (73.5–106.6)	22.89 (7.33–68.31)	8.32 (3.71–10.41)	20.34 (7.34–34.12)

### Expression and localization of YAP in human kidney biopsy samples

**Figure 6:**
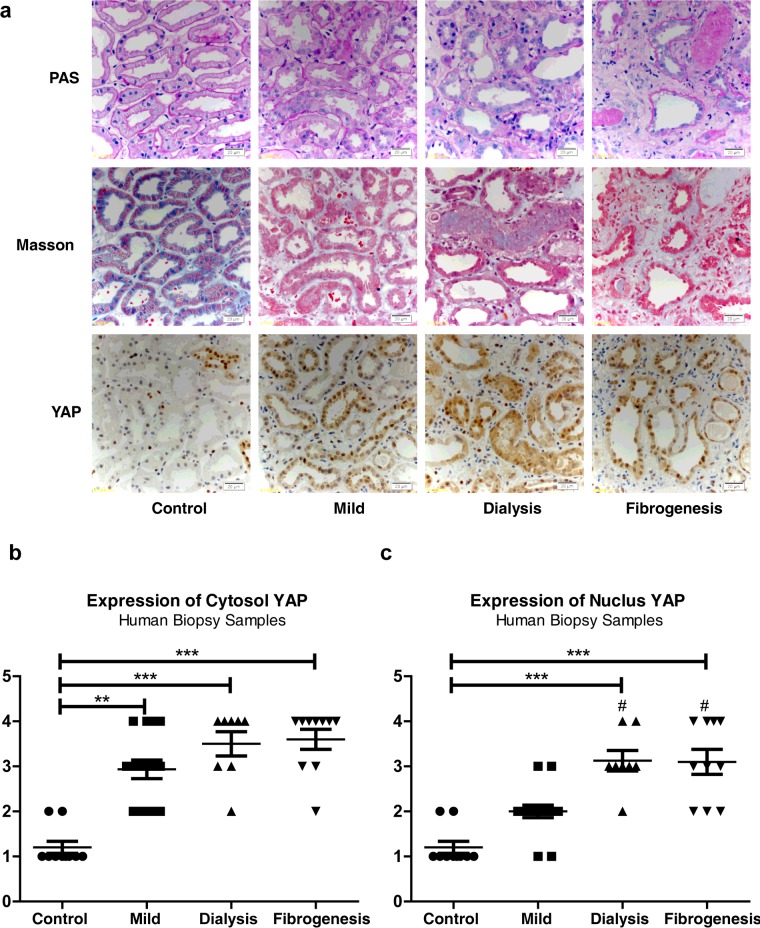
Pathological features and YAP immunostaining in AKI human biopsy samples (**a**) PAS staining (×400 magnification) showing morphology changes in AKI groups. Scale bar, 20 μm (top). Masson's trichrome staining (×400 magnification) showing fibrosis as blue appearance. Scale bar, 20 μm (middle). YAP immunostaining (×400 magnification). Scale bar, 20 μm (bottom). (**b**) Cytosolic and nuclear YAP expression in different AKI groups compared with controls. **P<0.01 compared with control; ***P<0.001 compared with control; ^#^*P*<0.05 compared with mild-regenerating group.

We verified YAP expression in the kidney biopsy samples of patients with AKI. A total of 33 patients were recruited. Of these patients, 23 were included in the regenerating group and ten were included in the fibrogenesis group. Table 2 summarizes the demographic features of the patients and the ten control subjects with minor lesions or mild mesangial proliferation. AKI of these patients was caused by ischaemia, drug-induced nephrotoxicity and rhabdomyolysis. The patients in the fibrogenesis group were older than those in the mild-regenerating group (*P*=0.028). Masson's trichrome staining showed that collagen deposition increased in the kidney tissue of patients in the fibrogenesis group compared with the control group (Figure 6a). PAS staining defined the loss of brush borders in the renal tubules in regenerating and fibrogenesis groups. The expression of YAP in the control subjects was also relatively low (Figures 6a, 6b and 6c). Cytoplasmic YAP significantly increased in the mild-regenerating group compared with the control group. YAP in the cytoplasm and the nucleus also increased in the dialysis regenerating and fibrogenesis group compared with the control subjects. Nuclear YAP significantly increased in these two groups compared with the mild regenerating subgroup (Figures 6a, 6b and 6c). These results are consistent with our findings in the rat models. Therefore YAP is likely to be involved in regenerating and fibrotic kidneys after acute I/R injury occurs.

## DISCUSSION

In contrast with the heart and the central nervous system, which exhibit limited capability of regeneration after initial injury, kidneys can undergo dramatic recovery [5,6,32]. Successful regeneration requires rapid replacement of damaged tubular epithelial cells and reconstitution of normal tubular function. However, maladaptive repair results in progressive fibrotic chronic kidney disease [1–3]. Studies have revealed the relationship between tubular epithelial cells and kidney fibrogenesis, indicating that these factors are implicated in the AKI–CKD transition [12,23,26,33]. Nevertheless, the underlying mechanism remains unclear.

The present study identified the involvement of the Hippo pathway in the pathophysiological process of I/R-induced AKI, particularly during repair. The pattern of changes in the complete (I/R 30 min) repair model differed from that in the incomplete (I/R 45 min) repair model. Although the expression of Mst1, Lats1 and YAP changed in the same manner, YAP was probably suppressed by upstream pMst1 and pLasts1 and retained in the cytoplasm in the phosphorylated and deactivated form. In fact, the activation of YAP significantly increased after I/R injury occurred, as indicated by the decreased relative ratio of pYAP to total YAP. The activation of YAP was more prominent and remained significant until 4 weeks in the I/R 45 min group.

We evaluated the change in YAP-related proteins. The reported YAP downstream target CTGF and the two YAP partners TEAD2 and TEAD3 changed in a similar manner to YAP. In contrast, expression of the YAP-competing protein Vgll4 changed in an opposite manner. These results implicate YAP in the regulation of I/R-induced AKI, and the activation of the core upstream kinases of the Hippo pathway may occur as feedback reactions.

Further analyses of differentiation markers showed that vimentin, αSMA and PCNA expression changed in a similar pattern to YAP. By contrast, the quiescent polarized cell markers such as Na^+^/K^+^-ATPase at the basolateral side and AQP1 at the apical side decreased during repair. The level of these two proteins did not return to control levels in the severe I/R group after 4 weeks of reperfusion, and YAP remained at a high level. IHC analysis showed further that the majority of YAP was detected in the cytoplasm and nuclei of tubular epithelial cells. Serial sections revealed that YAP was strongly expressed in the cytosol and nuclei of the epithelial cells of the deformed tubules. YAP was also co-expressed with dedifferentiation and proliferation markers, including vimentin, pSmad2/3 and PCNA, but was changed inversely with mature cell markers. These findings indicated that YAP might be relevant to the proliferation and redifferentiation of the reconstituted epithelia after acute I/R injury. Thus this mechanism might be related to the AKI–CKD transition.

*In vitro* overexpression and RNAi analyses suggested that YAP could be involved in the proliferation, pro-fibrogenesis and dedifferentiation of HK-2 cells, which are promoted further by the nuclear-localized mutants S127A and 5SA. After the YAP agonist digitoxin was administered, proliferation was promoted, redifferentiation was prevented and fibrosis was exacerbated in the kidneys of I/R-induced AKI rat models. These experiments suggest that YAP might exhibit bidirectional functions in I/R AKI. During repair, YAP mainly exerted beneficial effects on the proliferation of the injured renal tubular epithelial cells. In contrast, constant increase and activation of YAP could exacerbate kidney fibrosis and impede the redifferentiation of dedifferentiated tubular cells.

We evaluated YAP expression in human AKI biopsy samples with different degrees of severity. YAP in the cytosol and nucleus increased in the mild and dialysis subgroups and in the fibrogenesis group compared with the control group, which exhibited low YAP expression. Moreover, YAP signal significantly increased in the nuclei of cells in the dialysis and fibrogenesis groups compared with the mild subgroup. Thus YAP activation increased in these two groups.

The involvement of YAP in tissue repair and regeneration has been observed in other organs in mammals [16–20,26]. YAP mainly elicits beneficial effects on myocardial ischaemic injury [34]. In mice suffering from myocardial infarction, cardiomyocyte-specific inactivation of YAP increases myocyte apoptosis and fibrosis [20]. Conversely, restoration of YAP activity decreases infarct size and improves cardiac function and survival [35–37].

The function of YAP has also been evaluated in organs similar to kidneys, such as the liver and small intestine. In a rat partial hepatectomy model, the Hippo pathway was altered after partial hepatectomy was performed [19]. Increased YAP activation and nuclear localization, as well as decreased activation of its upstream regulatory kinases Mst1/2 and Lats1/2 were identified at 1 day. However, the nuclear YAP protein levels and the expression of YAP target genes returned to basal levels when the liver reached the pre-hepatectomy size, which occurs after 7 days. In chronic cholestatic liver diseases, a more active nuclear YAP is found in the bile ducts of liver samples from patients with primary sclerosing cholangitis and primary biliary cirrhosis, as well as in the whole liver of the BDL (bile duct ligation) mouse model [18,26]. Ablating *Yap* in the mouse liver compromises bile duct proliferation, enhances hepatocyte necrosis and suppresses hepatocyte proliferation after BDL through the down-regulation of survivin expression. Likewise, increased YAP levels have been identified in regenerating crypts in a DSS (dextran sodium sulfate)-induced colonic regeneration model [20]. Inactivation of YAP severely impairs intestinal regeneration after DSS is administered.

YAP is involved in the regulation of proliferation and differentiation of adult cells. The inactivation of the Hippo pathway induces dedifferentiation of adult hepatocytes into cells bearing progenitor characteristics [38]. YAP induction is also responsible for smooth muscle phenotypic modulation from a contractile phenotype to a synthetic state in rat aortic smooth muscle cells and murine arterial injury models [39]. During kidney regeneration, YAP probably performs beneficial and detrimental functions. Considering the difference between complete and incomplete repair of I/R AKI, we proposed that YAP activity should be controlled in tissue regeneration to successfully complete compensatory proliferation and thus replace injured cells. The increase in YAP should be inhibited promptly during regeneration to prevent pro-fibrotic potential and allow appropriate differentiation of renal tubules.

The reported YAP antagonists verteporfin and Vgll4-mimicking peptide, which elicit positive effects on tumour growth suppression in liver and gastric cancers, may cause anti-fibrotic effects in an incomplete repair I/R AKI model [30,40]. Unfortunately, these agents were not available in the present study. We used dobutamine, which can stimulate YAP translocation from the nucleus to the cytoplasm in an osteosarcoma cell line and significantly suppress YAP–TEAD complex-mediated gene transcription [21]. However, the administration of the medicine for 28 days after I/R 45 min AKI induction did not improve the lesions in the incomplete repair models or delay the process of regeneration (results not shown). The proper modulation of YAP levels to improve renal tubule differentiation and prevent fibrogenesis might occur more efficiently by targeting its upstream regulatory proteins in addition to simply changing its phosphorylation. Kidney conditional *Yap*-knockout transgenic mice were also not available in the present study. Nevertheless, the precise stage at which YAP is specifically knocked out after the regeneration stage of I/R AKI may be difficult to achieve using the present transgenic mouse system. On the basis of all of the evidence we have observed, the regenerative effect of YAP in this model still has to be confirmed in future study.

In conclusion, the Hippo pathway is involved in the regeneration and fibrogenesis of kidneys after acute I/R injury. YAP also performs proliferative and pro-fibrotic functions during the recovery. A constant increase and activation of YAP may be related to interstitial fibrosis and abnormal differentiation of renal tubules. Thus the appropriate modulation of this protein during repair may be a potent therapeutic target in the AKI–CKD transition after I/R injury occurs.

## Abbreviations

AKI: acute kidney injury
AQP1: aquaporin 1
BDL: bile duct ligation
CKD: chronic kidney disease
CTGF: connective tissue growth factor
DSS: dextran sodium sulfate
E-cadherin: epithelial cadherin
GAPDH: glyceraldehyde-3-phosphate dehydrogenase
H&E: haematoxylin and eosin
IHC: immunohistochemistry
I/R: ischaemia/reperfusion
Lats1/2: large tumour suppressor 1/2
Mst1/2: mammalian sterile 20-like kinase 1/2
PAS: periodic acid–Schiff
PCNA: proliferating-cell nuclear antigen
RT: reverse transcription
αSMA: α-smooth muscle actin
TEAD: TEA domain family member
Vgll4: vestigial-like protein 4
YAP: Yes-associated protein
